# Histopathological Findings in Testes from Apparently Healthy Drones of *Apis mellifera ligustica*

**DOI:** 10.3390/vetsci7030124

**Published:** 2020-09-02

**Authors:** Karen Power, Manuela Martano, Gennaro Altamura, Paola Maiolino

**Affiliations:** Department of Veterinary Medicine and Animal Productions, University of Naples “Federico II”, Via Delpino, 1, 80137 Naples, Italy; manuela.martano@unina.it (M.M.); gennaro.altamura@unina.it (G.A.); maiolino@unina.it (P.M.)

**Keywords:** histopathology, honeybee, testes

## Abstract

It is well known that factors acting on the decrease of population of honeybees, can act on the male and female reproductive system, compromising the fertility of queens and drones. While there are many studies on female fertility, only a few studies have focused on male fertility and the possible alterations of the reproductive system. The testes of 25 samples of adult drones of *Apis mellifera ligustica* were analyzed by histopathology using an innovative histological processing technique and the alterations that were found are here described. Most of the samples showed unaltered testes but, in some cases, samples showed degenerated seminiferous tubules, while others appeared immature. Although a limited number of samples were analyzed, the results obtained displayed that histopathological alterations of the testes exist also in honeybees and that more interest should be put to the matter, as honeybees could be considered as bioindicators for endocrine disruptors. Future studies on a larger number of samples are necessary to analyze how different environmental factors can act and induce alterations in the honeybee reproductive system.

## 1. Introduction

Honeybees are of proven importance for the agricultural economy and the conservation of biodiversity [1], as given by their global distribution and generalist foraging behavior. Honeybees can be considered the most important single pollinator species of a wide variety of wild flora, livestock pastures, and private gardens [2,3]. Pathogens, agrochemicals, and climate alterations are only a few of the environmental biotic and abiotic elements that, acting singly or synergistically, are able to threaten the fitness of honeybees and lead to colony losses [4,5]. When a drastic reduction in the number of individuals of a species occurs, effective reproduction is potentially the key to perpetuation and conservation of the species. Ineffective reproduction, caused by numerous factors such as heavy metals, chemicals, and diseases, could lead to a reduction in the offspring, increasing the risk of a further decrease in the population [6]. The stressors mentioned above are able to directly or indirectly have pathological effects on the reproductive system of honeybees, impairing their fertility and often diminishing the number of future offspring that should perpetuate the species [7,8,9]. To date, many studies have focused on alterations of queen fertility, as outputs, such as low egg deposition or a high prevalence of drone brood, are easily recognizable in hives [10]. On the contrary, hypofertility of drones is often subclinical and therefore less studied, but, as the reproductive activity is strictly connected to the success of mating, all the elements that could invalidate drones’ fertility, could consequently threaten colony fitness [11].

In colonies of *Apis mellifera*, drones typically represent 5 to 10% of the adult population, however the production and maintenance are regulated by the colony in accordance with several environmental factors, namely food availability, size of colony, number of drones already present in the colony, queen presence/absence, and season [12]. The main role of drones in the colony organization is to reach the Drone Congregation Areas (DCAs) and mate with queens from other colonies, spreading the genetic material of the colony from which they belong to new colonies [12]. Therefore, ability to fly, copulate, and a great amount of high-quality sperm, are required to fulfill the precious role of drones [13].

The reproductive process in honeybees shows unique features as queens only mate in the early stages of their life with multiple drones and acquire on that occasion the whole amount of spermatozoa that will be stored in the spermatheca and used during their whole life [14]. Therefore, drone fertility is strictly connected to the queen’s reproductive capacity as mating with unfit drones, which are not able to produce high-quality fertile semen, could lead to queen failure, which occurs when queens stop efficient egg laying or start laying haploid male eggs [10]. Queen failure causes queen replacement by colony workers or by beekeepers, with a consequent increase in production time and costs [15].

The male reproductive system of *A. mellifera* consists of a pair of bean-shaped testes, composed of 150 or more seminiferous tubules per testis, from which originate two vasa deferentia that enlarge distally forming the seminal vesicles, which open in the mucus glands [16]. Mucus glands are elongated accessory glands that produce white mucus, a proteic substance used to produce a mating sign in the queen after successful copulation [17]. The mucus glands open into paired, lateral ejaculatory ducts, which in turn convey into a long, slender, common ejaculatory duct. The common ejaculatory duct opens in the bulb of the endophallus, the copulatory organ. The honeybee copulatory organ is located internally, in the ventral region of the abdomen and it is composed of three main elements: the bulb, provided with the chitinous plates, the cervix, and the vestibulum, presenting two yellow cornua.

Two more accessory sex glands, found near the endophallus bulb, are recognized: the bulbous gland [16], and the cornual glands. The cornual glands secrete an orange-colored secretion that reinforces the attachment of the mating sign in the queen’s reproductive tract [18].

In *A. mellifera* drones, the formation of the male reproductive system starts during the first stages of embryonic development. Testes are formed soon before the larva hatches from the egg while spermatogenesis starts on the third day of the larval stage. Spermatogonia, the undifferentiated germ cells, undergo multiple mitoses, developing in primary spermatocytes [16]. The primary spermatocytes are subjected to a reductional meiosis where a secondary haploid spermatocyte and one cell containing only cytoplasm, are formed. The secondary spermatocytes then undergo a non-reductional meiosis, giving origin to two spherical spermatids. Spermatid multiplication stops prior to pupation [16] and honeybee drones seem to be the only insects in which spermatogenesis occurs only during their developmental stages, therefore, drones have a predetermined quantity of sperm in adult life [19,20]. Spermiogenesis, which is the morphological differentiation resulting in spermatozoa maturation, starts two to three days after pupal molting, and sperm migrate from the testes to the seminal vesicles, where they absorb nutrients to become fully functional [21]. Drones are sexually mature twelve days after emergence when they are able to evert their endophallus and creamy colored semen containing spermatozoa, which can be located at the posterior extremity of the ejaculate, on top of the white mucus, which is void of spermatozoa [17]. Drones produce an average of 1.5 to 1.7 μL semen with approximately 7.5 million spermatozoa/μL [22].

Considering the peculiarity of the male reproductive system development, factors affecting the larval and pupal stages are potentially able to impair the whole reproductive life of a drone.

Colonies in which a poor protein diet was fed, raised drones with lower body and thorax mass and lower ejaculate volumes, compared to colonies in which pollen administration was guaranteed [23]. On the other hand, by feeding colonies with sucrose syrup and protein supplements during the early spring, the semen quality improved [24].

It has been proven that low levels of *Varroa destructor* during drone pupal development can directly cause flight reduction and reduce sperm production down to 45%, making them unlikely to reach the DCAs, chase the queen, copulate with her, and transfer a sufficient amount of spermatozoa [25].

However, it has also been shown that drones infected by *Nosema apis* during adult life, particularly shortly after hatching, face substantial fitness costs that impair the production and maintenance of high-quality sperm, reducing fertility [26].

An active debate is still open on the influence of age on sperm quality, quantity, and viability: some have shown that sperm viability decreases with age [27], while others observed an increased viability with age [28]. According to Rousseau et al. [22] age has no effect on spermatozoa viability and motility, while for Stürup et al. [11] senescence negatively influenced sperm viability only from 20 to 25 days after emergence, but the length of viability decrease is influenced by colony factors, especially genetics.

Previous studies have shown that various pathogens can be found in the reproductive organs of drones [26,29] and that stressors like pesticides [7], miticides [30], and high temperatures [11] can directly impair drones’ fertility by reducing spermatozoa concentration, viability as well as ATP concentration, necessary for spermatozoa motility [8].

To date, many studies have focused on the viability and motility of spermatozoa, given the increasing interest of beekeeping in instrumental insemination, and little importance has been given to the study of possible alterations of spermatozoa [31] and not much is known about pathological changes in the microscopic structure of reproductive organs which could lead to the formation of altered spermatozoa.

The aim of this preliminary study was to analyze, through the use of an innovative histologic processing technique, the testes of apparently healthy *Apis mellifera ligustica* drones collected in different apiaries across the Campania region (Italy) and to describe the presence of the alterations unexpectedly found.

## 2. Materials and Methods

Twenty-five adult drones of *A. m. ligustica* were collected in 5 different apiaries located in Campania (Italy) (5 drones/apiary) from March to June 2019, a time-span that includes the natural reproductive season of local honeybees. Apiaries were located in small beekeeping farms (less than 30 hives) surrounded by orchards and tomato crops. Drones were individually collected from apparently healthy hives, with low levels of *V. destructor* infestation (<2%) and absence of clinical signs of viral and *N. apis* infection. Insects were selected according to size, vitality, and absence of visible clinical signs of pathologies such as trembling, deformed wings, and swollen abdomens. They were manually caught from the comb and subsequently transported in sterile tubes to the laboratory of Veterinary General Pathology and Anatomical Pathology of the Department of Veterinary Medicine and Animal Productions, University of Naples “Federico II”. All drones were anesthetized with chilling for 3 min at −20 °C [32], observed at the stereomicroscope (Zeiss Stemi 305 trino, New York, NY, USA) to detect the presence of macroscopic alterations, and whole-body mass was weighted to assess age according to Metz and Tarpy [13]. Samples were then placed in tubes containing 10% buffered formalin for 1 h for histopathological examination. Subsequently, they were individually injected with 10 μL of 10% buffered formalin using a micropipette (Accumax Smart, Dantali, India) and a 10 μL tip. While holding the thorax of the drone between the thumb and the index finger of one hand, the injection was performed laterally between the 3rd and 4th tergite, holding the tip parallel to the tergite in order to avoid puncturing of the gut and contamination of specimens with pollen and feces [32]. After injection, drones were moved to a tube containing 10% formalin. After 24 h, each sample was cut lengthwise, placed in an embedding cassette, and then in an automatic embedding processor (VTP300, Bio-Optica, Milan, Italy). For each half, paraffin blocks were manually created using an embedding console system, and 3 μm thick sections were obtained with a microtome. In order to facilitate sectioning, a disposable stainless-steel blade (N35, Feather, Osaka, Japan) for fine cuts of hard tissues was used. Single sections were placed on the surface of hot water and then collected on a slide and dried at room temperature for 12 h. Slides were mechanically stained with hematoxylin and eosin (H-E) using an automatic tissue slide stainer (ST5010 Autostainer XL, Leica, Germany) and finally mounted. Tissue preparations were observed by light microscopy (Nikon Eclipse E-600, Nikon, Tokyo, Japan). Although observation focused on the testes, all tissues were analyzed for the presence of visible pathogens, i.e., *Nosema* spp.

## 3. Results

**Figure 1:**
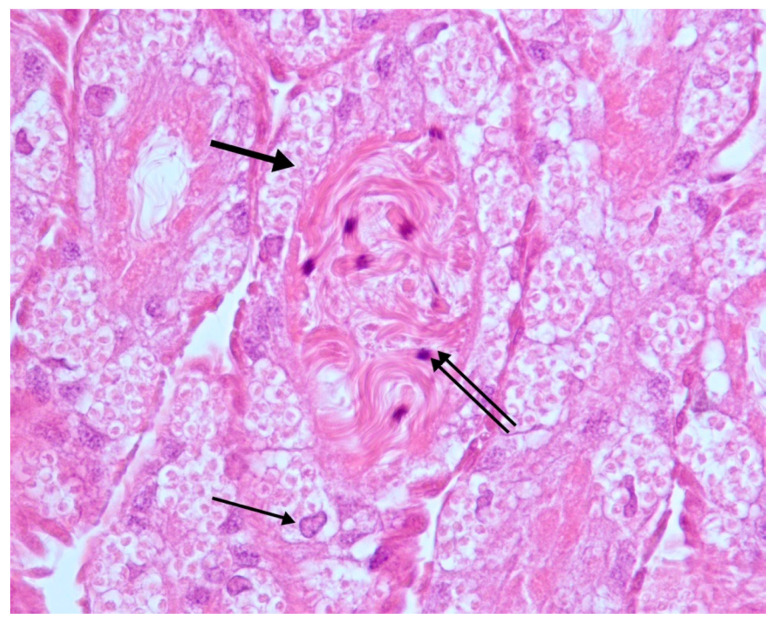
Testes. Normal seminiferous tubules. Follicular (thin arrow) and germ cells (thick arrow); coiled spermatozoa in the lumen of the tubules (double arrow). H-E 40×.

Macroscopically, samples showed no visible alterations and were aged 10 to 27 days old. Samples processed with the protocol described above appeared well preserved since tissues were well stained, and no artifacts were seen. Microscopically, 17/25 samples showed healthy testes that appeared as elongated, bean-shaped structures with numerous seminiferous tubules, surrounded by an external epithelial layer (seminiferous epithelium). The seminiferous tubules contained follicular and germ cells that encapsulate to form a thin wall cyst; the tubular lumen was filled with many coiled spermatozoa (Figure 1).

**Figure 2:**
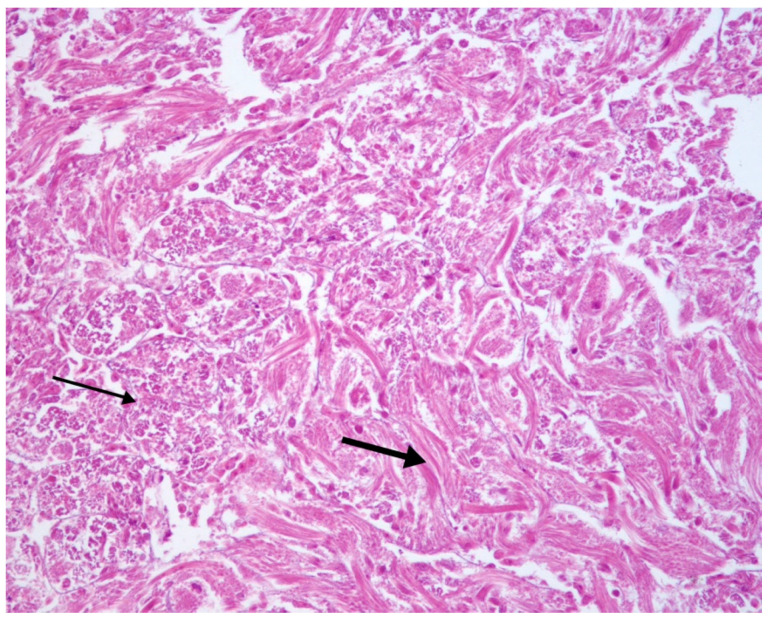
Testes. Altered seminiferous tubules. Disappearance of the seminiferous epithelium and the lumen; necrosis of follicular and germ cells (thin arrow); numerous spermatozoa (thick arrow); absence of spermatocytes and spermatogonia. H-E 20×.

In 5/25 samples, the seminiferous tubules presented severe and widespread degenerative phenomena characterized by the appearance of necrosis of follicular and germ cells, disappearance of the external epithelial layer and of the tubular lumen, until the complete disruption of the normal tissue structure. It was possible to distinguish numerous spermatozoa and the complete absence of spermatogonia and spermatocytes (Figure 2).

**Figure 3:**
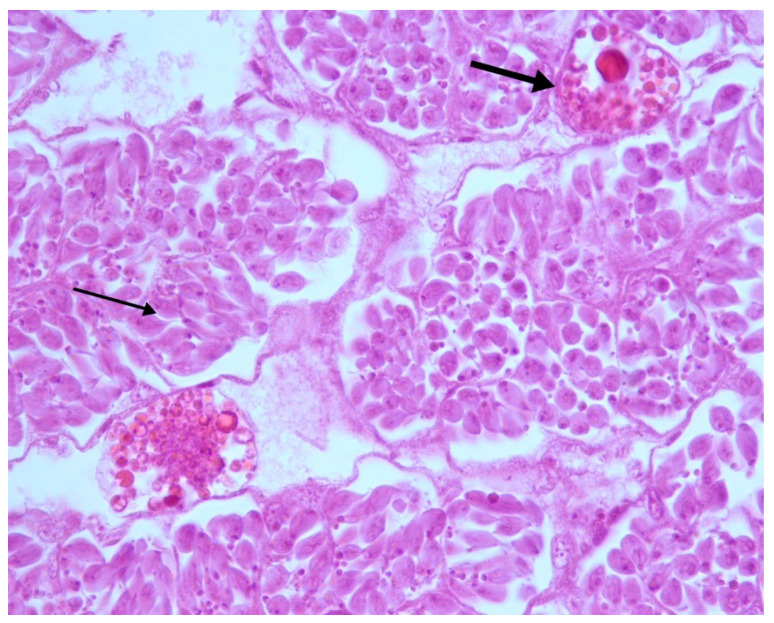
Testes. Altered seminiferous tubules. Absence of tubular lumen; presence of numerous spermatogonia (thin arrow); absence of spermatozoa; trophocytes between the tubules (thick arrow). H-E 40×.

In 2/25 samples, the seminiferous tubules were characterized by a small number of tubules of reduced dimensions, absence of tubular lumen and numerous spermatogonia but no spermatozoa, suggesting they had not reached complete maturation. Between the tubules it was possible to observe the presence of trophocytes and eosinophilic granules, attributable to residues of trophocytes (Figure 3).

**Figure 4:**
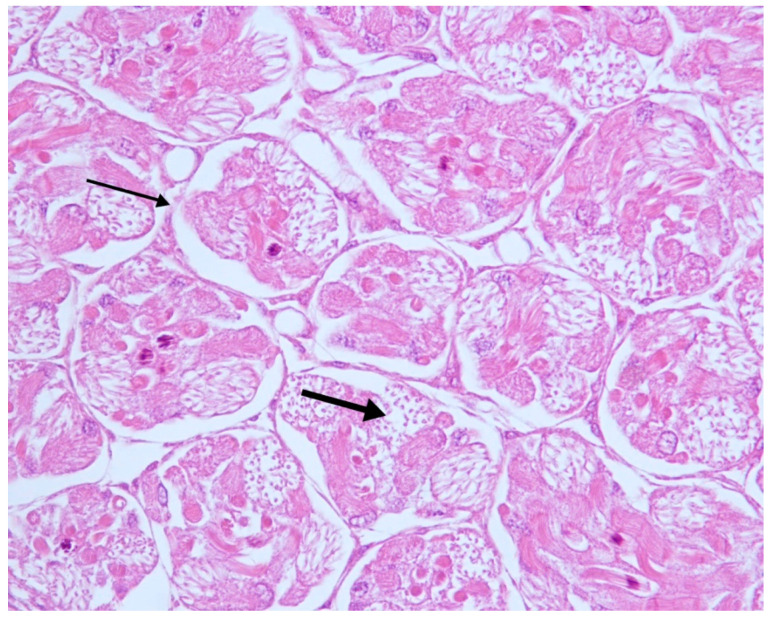
Testes. Altered seminiferous tubules. Detachment of germ cells from the basal lamina (thin arrow); rupture of the membranes of spermatogonia (thick arrow). H—E 20×.

In 1/25 samples it was possible to observe the detachment of the germ cells from the basal lamina of the tubules and the rupture of the membranes of the spermatogonia, probably a consequence of severe and diffuse intratubular edema (Figure 4).

## 4. Discussion

Compared to the past, in recent years, male hypofertility/infertility has been arousing greater interest, since malformations, genetic alterations, infectious diseases, food shortages, and managerial errors have been identified as responsible for a decreased reproductive capacity of many zootechnical species [33,34]. In a previous study [35], we had highlighted the existence of spermatozoa showing visible defects such as broken, split, and double tails. In this study, we describe the presence of alterations of the reproductive system in *A. m. ligustica* drones, regardless of the absence of macroscopic alterations, and we suggest that altered testes could probably be the cause of altered spermatozoa, that are unfit to swim up the oviducts, reach the spermatheca, and subsequently fertilize the egg. Therefore, a queen who would have mated with these drones would have very likely preserved in her spermatheca fewer or abnormal spermatozoa, reducing the potential to lay diploid eggs.

The presence of alterations in apparently healthy drones becomes particularly relevant for instrumental insemination. Considering the peculiar reproductive behavior of honeybees, instrumental insemination is a valuable tool to control the source of males and avoid undesired mating with drones that could negatively influence the genetics of the colony. Donor drones are chosen mainly on genetic characteristics and the absence of clinically visible signs of disease, while actual health status and semen analysis is not always performed prior insemination. Unhealthy and unfertile semen can, therefore, erroneously be used causing a reduction in queens’ reproductive performance, as well as disease spreading.

Most of the samples did not show any pathological alterations, however, conversely to previous descriptions, the lumen of the seminal tubules appeared filled with coiled spermatozoa. Spermatozoa maturation and migration to the seminal vesicles have been often described as completed during the first week of adult life [16,19], however, empirical data supporting this theory appears old and limited, and no histological study has ever been performed, therefore comparable results are currently unavailable. On the contrary, a study by Metz and Terpy [13] found that the transfer of spermatozoa from the testes to the seminal vesicles can actually begin in the first week, but no data is reported for the end time of the migration.

Five samples showed clear degenerative phenomena affecting the seminiferous tubules. In other species, degenerative alterations have been associated with high levels of heavy metals and pesticides, such as organophosphates or neonicotinoids [36,37], in the environment which could induce oxidative stress in tissues. Oxidative stress occurs following the accumulation in organisms of reactive oxygen species (ROS) which can determine high molecular damage, degeneration of tissues, and premature aging [38]. It has been shown that drones are able to survive acute oxidative stress due to individual tolerance and resistance, and not to repair of oxidative damage of lipids and of cells [39], thus leading to a subclinical disease.

For this study, only adult drones ready for mating were collected. Nonetheless, the microscopic examination highlighted samples with testes that showed reduced maturation, as can be inferred from the presence of many spermatogonia and degenerated trophocytes in the intertubular space.

Testis development and spermatogenesis of drones of *A. mellifera* have been precisely described by Lago et al. [40] based on histological sections. Changes in whole testicular architecture, as well as of the seminiferous tubules, are described from the first-instar larvae to the pharate-adult stage. According to the histological descriptions, our findings correspond with changes occurring in testes of a fifth-instar stage larvae, however, the presence of trophocytes is not described in the cited research.

Neonicotinoids and other pesticides are considered as endocrine disruptors able to induce both hormonal and morphological alterations of the reproductive system, by mimicking the effects of estrogens and inducing signs of feminization and demasculinization [41,42]. Although endocrine disruptors are found in minimal quantities, in the long run, their chronic accumulation can interfere with honeybee health and the correct function and development of the reproductive system of drones [43,44]. Furthermore, endocrine disruptors seem to induce the production of vitellogenin (Vg) in male specimens of many animal species, and probably also in honeybees [45]. Vg is a protein present in the fat body and in the hypopharyngeal glands of workers, queens, and drones which plays a key role in phenomena related to egg laying, immunity, and longevity [46,47]. It cannot be excluded that in the drones analyzed in this study there may be an up-regulation of Vg during the developmental stages which could have influenced the correct maturation of the male reproductive system or induce alterations.

Degeneration or delayed or incomplete maturation could also have been related to the presence of subclinical viral and parasitic diseases.

Deformed Wing Virus (DWV) as well as *N. apis* have been localized in the testes of mature drones [26,29] suggesting a possible action of these pathogens in drones’ fertility impairment although no histopathological findings in the reproductive tissues have been described in previous studies.

In the present study, drones did not show any clinical signs of either disease, but, while we can exclude the presence of *N. apis* and *Nosema ceranae*, as no spores were identified in the gastrointestinal tissue neither in the reproductive tissue, the presence of low levels of DWV cannot be excluded.

Samples were collected from colonies infested with low infestation levels of *V. destructor*, which, as previously stated is correlated with a low number of spermatozoa [25] and oxidative stress [48] but also to the spreading of DWV [49].

## 5. Conclusions

In conclusion, the results obtained, although carried out on a limited number of samples, have allowed us to display that the morphological alterations of the testes also exist in honeybees and that these could cause the formation of altered spermatozoa. Moreover, we have shown that the macroscopic appearance does not always reflect the actual health status of drones and this information appears particularly important when selecting donors for instrumental insemination, therefore, semen analysis should always be performed. We can also hypothesize that the alterations found here can be ascribed to the same causes as those in humans and other animals. Unfortunately, due to the techniques used to process the samples (formalin fixation and paraffin embedding) and to the unexpectedness of the results, it was not possible to use the same samples for further studies and correlate a specific cause for the histological alterations found. Therefore, more studies are needed to identify the etiology of testicular lesions. If the hypothesis of a major role of endocrine disruptors should be confirmed, honeybees could have the potential of becoming bioindicators of the presence of endocrine disruptors in the environment that could also affect fertility in male humans. The alterations found in honeybee testes and spermatozoa could be a red flag for similar issues affecting humans.
